# Development of a real-time recombinase-aided amplification assay for rapid and sensitive detection of *Edwardsiella piscicida*

**DOI:** 10.3389/fcimb.2024.1355056

**Published:** 2024-03-28

**Authors:** Yuchen Dong, Dandan Zhou, Binzhe Zhang, Xiaoying Xu, Jian Zhang

**Affiliations:** ^1^ School of Ocean, Yantai University, Yantai, China; ^2^ Yantai Marine Economic Research Institute, Yantai, China

**Keywords:** *E. piscicida*, aquaculture, recombinase-aided amplification, rapid diagnosis, isothermal amplification

## Abstract

*Edwardsiella piscicida*, a significant intracellular pathogen, is widely distributed in aquatic environments and causes systemic infection in various species. Therefore, it’s essential to develop a rapid, uncomplicated and sensitive method for detection of *E. piscicida* in order to control the transmission of this pathogen effectively. The recombinase-aided amplification (RAA) assay is a newly developed, rapid detection method that has been utilized for various pathogens. In the present study, a real-time RAA (RT-RAA) assay, targeting the conserved positions of the *EvpP* gene, was successfully established for the detection of *E. piscicida*. This assay can be performed in a one-step single tube reaction at a temperature of 39°C within 20 min. The RT-RAA assay exhibited a sensitivity of 42 copies per reaction at a 95% probability, which was comparable to the sensitivity of real-time quantitative PCR (qPCR) assay. The specificity assay confirmed that the RT-RAA assay specifically targeted *E. piscicida* without any cross-reactivity with other important marine bacterial pathogens. Moreover, when clinical specimens were utilized, a perfect agreement of 100% was achieved between the RT-RAA and qPCR assays, resulting a kappa value of 1. These findings indicated that the established RT-RAA assay provided a viable alternative for the rapid, sensitive, and specific detection of *E. piscicida*.

## Introduction

*Edwardsiella piscicida*, previously known as *E. tarda*, is a Gram-negative, rod-shaped, facultatively anaerobic, intracellular pathogen, which is widely prevalent in aquatic environments and causes systemic infections in various species such as fish, amphibians, reptiles, and mammals (Leung et al., 2019). Moreover, *E. piscicida* also serves as an etiological agent for both extraintestinal and gastrointestinal infections in humans (Leung et al., 2019). The initial documentation of *E. piscicida* infection in aquaculture was observed in channel catfish (Meyer and Bullock, 1973), subsequently, severe infections caused by *E. piscicida* have been reported in numerous commercially significant marine and freshwater fish species, including eels, mullet, chinook salmon, Japanese flounder, turbot, tilapia, and striped bass, leading to large economic losses in aquaculture worldwide (Park et al., 2012). Similar to other invasive pathogens, *E. piscicida* exhibits a preference for infiltrating host cells as a means to evade immune system detection and facilitate replication within both phagocytes and nonphagocytes, thereby contributing to its pathogenicity (Xie et al., 2014; Zhang et al., 2016). In recent years, increasing number of *E. piscicida* isolates have been obtained from aquacultural environment (Ferrari et al., 2022; Kim et al., 2022; Sugiura et al., 2022). Consequently, the development of a prompt and highly sensitive diagnostic technique for *E. piscicida* is of utmost importance in the prevention of outbreaks.

Currently, the identification of *E. piscicida* primarily depends on conventional techniques, including culture, PCR, or immune-testing (Swain et al., 2001; Hong et al., 2009; Castro et al., 2010; Ferrari et al., 2022; Sugiura et al., 2022). While cultures offer undeniable proof of infection, their lengthy incubation period renders them impractical for clinical diagnosis, except for antimicrobial susceptibility analysis (Kim et al., 2022; Sugiura et al., 2022). Various methods that rely on antibodies, such as enzyme-linked immunosorbent assay (ELISA), and quartz crystal microbalance (QCM) biosensor, have been developed for *E. piscicida* detection (Swain et al., 2001; Hong et al., 2009). Over the past few years, several PCR-based molecular detection systems, and loop-mediated isothermal amplification (LAMP) have been developed for detection of *E. piscicida* (Castro et al., 2010; Xie et al., 2012; Reichley et al., 2015). However, the complex operation or expensive equipment requirements limit their application in clinical or environmental samples. Thus, it is necessary to establish a fast, simple, and sensitive method for *E. piscicida* detection.

The recombinase-aided amplification (RAA) assay is an innovative isothermal amplification technology, which offers distinct advantages such as quickness (within 20 min), simplicity, and low cost (Pang and Long, 2023). Consequently, RAA surpasses other methods and holds great potential for pathogen diagnosis (Fan et al., 2021). The RAA reaction system consists of three essential proteins: recombinase UvsX, which facilitates the annealing of primers to template DNA; single strand DNA binding protein (SSB); and DNA polymerase, which enhanced the speed and specificity nature of the reaction system. In recent time, RAA has proven effective in identifying different types of microbial pathogens (Wang et al., 2020; Qin et al., 2021; Tu et al., 2021).

The gene *EvpP* (*E. tarda* virulent protein P) is an important T6SS effector, which is essential for the virulence of *E. piscicida* (Hu et al., 2014). Interestingly, *EvpP* gene could not be found in other bacteria with T6SS (Ho et al., 2014). In this study, we effectively developed a real-time RAA (RT-RAA) based method for detecting *E. piscicida*, specifically targeting the *EvpP* gene. This assay exhibits accessibility, specificity, and sensitivity, with a completion time of just 20 minutes. The characteristics of the RT-RAA method make it a promising candidate for early diagnosis of *E. piscicida* infection.

## Material and methods

### Bacteria and clinical and environmental samples

*Edwardsiella piscicida* DC1 was isolated from diseased turbot (*Scophthalmus maximus*) reared in a commercial fish farm located in Qingdao, Shandong province, which also showed high pathogenicity to *Lateolabrax maculatus*. Another eight clinically-common pathogens were evaluated in this study, including *Pseudomonas fluorescens*, *Leclercia adecarboxylata*, *Vibrio rotiferianus*, *Vibrio scophthalmi*, *Vibrio alginolyticus*, *Vibrio hyugaensis*, *Haemophilus piscium* and *Vibrio azureus*, which were all isolated and stored in our lab. All strains were verified by 16S rDNA gene sequencing in Sangon Biotech Co., Ltd. (Shanghai, China). All strains were cultured in a Luria-Bertani broth (LB) medium at 28°C.

For clinical specimen preparation, 30 clinical samples were prepared. Briefly, clinically healthy *L. maculatus* (average weight 14.7 g) were purchased from a commercial fish farm located in Yantai, Shandong province. Before experiment, fish were randomly sampled for the examination of *E. piscicida* presence by plate counts. For experimental infection, *E. piscicida* DC1 were cultured in LB medium to an OD_600_ = 0.8, *L. maculatus* were challenged via intraperitoneal (i.p.) injection with 100 μl of *E. piscicida* DC1 (10^7^ cfu/ml) and maintained at 22°C. At 0, 12, 24, 36, and 48 h post-challenge (hpc), the liver, spleen, and kidney were obtained aseptically from the fish (three fish/time point). For *E. piscicida* detection, the tissues obtained above were homogenated in PBS, spread onto the LB agar plates, and incubation at 28°C for 48 h, furthermore, the observed bacterial colonies were verified by 16S rDNA sequencing (Zhang et al., 2014). The samples were further confirmed by quantitative real-time PCR as reported previously (Reichley et al., 2015). A total of 24 positive and 6 negative fish samples were prepared for further RT-RAA analysis. For environmental samples preparation, nine sea water and nine sediment samples were collected from the intertidal zone of Yantai, China. Before experiment, these samples were randomly sampled for the examination of *E. piscicida* presence by plate counts. Different concentrations of *E. piscicida* cultured above were added, and verified as above. A total of 12 positive (6 sea water and 6 sediment) and 6 negative samples (3 sea water and 3 sediment) were prepared for further RT-RAA analysis.

### DNA extraction

For bacterial DNA isolation, 1 ml sample of pure cultured bacterial suspension was obtained. The DNA extraction was performed using the TIANamp Bacteria DNA Kit (Tiangen Biotech Co, Ltd, China) following to the manufacturer’s instructions. For clinical samples’ DNA extraction, 50 mg samples were homogenized in 200 μL PBS and the total DNA was extracted using CTAB method (Guerra et al., 2020). The DNA were eluted in 100 μL of nuclease-free water and stored at a temperature of -20°C until utilized.

### Recombinant plasmid construction

The *EvpP* gene was selected as a target gene for detecting *E. piscicida*. The *EvpP* gene sequence was obtained from *E. piscicida* DC1, and submitted into the GenBank (accession no. OR670479.1). Using primers EvpP-F1/R1 (**Table 1**), we amplified a 591 bp fragment of the *EvpP* gene and inserted it into the pMD-19T vector (TAKARA, Dalian, China). The recombinant plasmid was confirmed by sequencing in Sangon Biotech Co., Ltd. and named pEvpP. The recombinant plasmid’s concentration was quantified using Nano-500 micro-spectrophotometer (Allsheng, Hangzhou, China) and the DNA copy number was determined using the formula: DNA copy number (copies/μL) = [6.02 × 10^23^ × plasmid concentration (ng/μL) × 10^−9^]/[DNA length × 660]. Then, the standard recombinant plasmids diluted in a 10-fold manner ranging from 2.1×10^8^ to 2.1×10^0^ copies/μL and stored at −20°C until further use.

**Table 1:** List of optimal primers and probes.

Primer/probe	Sequence (5’- 3’)	Product size (bp)
F1	ATGCTACTGAAAAAAGGATGGCG	591
R1	CTATTTCAATATTGAAAATTGGTGGC	
F2	GATGGCGGTCTGCGCCAGCGTATGCTGATC	139
R2	TAAATCCACCGAACCAGGCATAGACGGGAA	
Probe-RAA^a^	ACGGGGTATCAATGAGGGAATGGGGACGAC[6-FAM-dT]CA[THF]C[BHQ1-dT]CCGTGATCAAAGAAA- C3 Spacer	
F3	GGGTATCAATGAGGGAATGGG	106
R3	CAGGCATAGACGGGAATCTTT	
Probe-qPCR^b^	[6-FAM]TTTCTTTGATCACGGAGGTGAGTCGTC[BHQ1]	

^a^Probe for RT-RAA assay; ^b^Probe for qPCR assay.

### RAA primer design

Other nine *EvpP* genes of different *E. piscicida* strains were obtained from the National Center for Biotechnology Information (NCBI) database. The DNAMAN software was utilized to conduct multiple sequence alignment in order to identify the conserved regions of the *EvpP* gene. The primers and probe for RT-RAA were designed within the highly-conserved regions using an online tool “Primer & probe design for RPA/RAA” (https://ezassay.com/primer), adhering to the guidelines for designing RT-RAA primers and probe. To ensure specificity, a Primer-BLAST search was performed against the NCBI database to verify the sequences the primer and probe. Furthermore, the possibility of primer dimers and hairpins was evaluated by utilizing the online OligoEvaluator software (http://www.oligoevaluator.com). Finally, the primer and probe were synthesized and purified using high-performance liquid chromatography in Sangon Biotech Co., Ltd.

### Screening the optimal primers for RT-RAA assay

**Figure 1:**
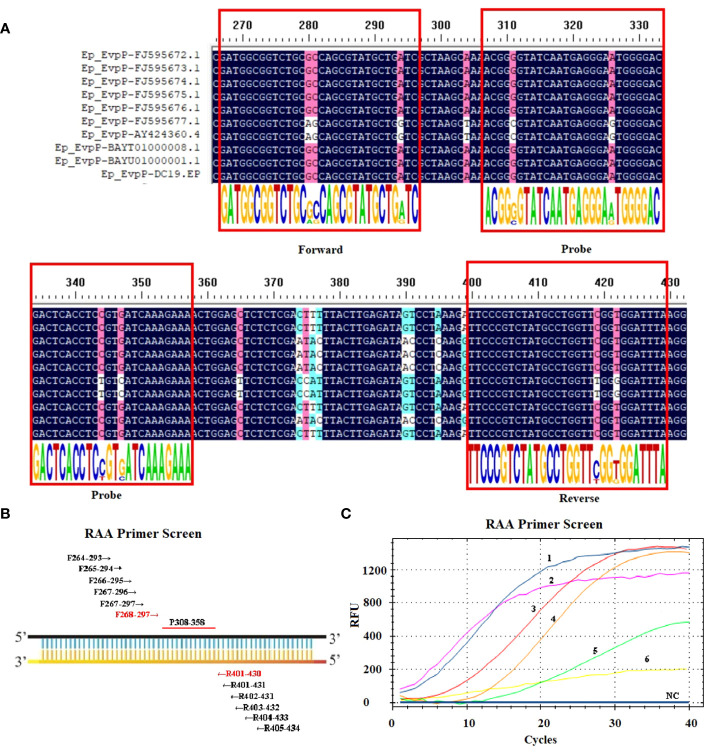
Screening the optical primers for RT-RAA assay. **(A)** Multiple sequence alignment of primers and probe with *EvpP* sequences of *E. piscicida* strains. The consensus residues are in black, and the residues that are ≥75% identical among the aligned sequences are in pink. **(B)** Sketch map of primary primers screening, the numbers indicate the position within the *EvpP* gene. **(C)** Screening results of the forward and reverse primers. 1: F268-297/R401-430; 2: F264-293/R405-434; 3: F266-295/R403-432; 4: F267-297/R401-431; 5: F267-296/R402-431; 6: F265-294/R404-433; NC, Negative Control.

First, an ideal probe (p308–358) was designed (**Table 1**, **Figure 1**). Then, six candidate primer pairs (F264–293/R405–434, F265–294/R404–433, F266–295/R403–432, F267–296/R402–431, F267–297/R401–431, F268–297/R401–430) were designed (**Supplementary Table S1**; **Figure 1**). The RT-RAA assay was performed with the above primers and the probe, and the primer pair with the highest end fluorescence intensity value (RFU value) and better “S” amplification curve was selected for further analysis. All reactions were performed three times.

### RAA assay

A commercial RAA fluorescence method kit (Zhongce, Hangzhou, China) was used to perform the RT-RAA assay in a reaction volume of 20 μL according to the manufacturer’ s instructions. Each primer and probe described above was diluted to a final concentration of 10 μM. The reaction mixture contained 2.0 μL of DNA template that was extracted, 10 μL of reaction buffer, 5.56 μL of DNase-free water, 0.4 μL of each primer, 0.24 μL of probe, 1.0 μL of magnesium acetate and reactive dry powder. Following the addition of all remaining components to the reactive dry powder, magnesium acetate is subsequently introduced to the tube lids. Subsequently, the tube lids are securely closed and the reaction tubes are subjected to gentle agitation by inverting them 5-6 times, followed by centrifugation at a low speed for a duration of 10 seconds. Afterward, the resulting tubes are transferred to the Bio-rad CFX96 Touch Real-Time PCR Detection System (Bio-rad, Hercules, CA, USA) and maintained at a temperature of 39°C for 20 minutes. Each experimental run includes both a positive control (pEvpP plasmid) and a negative control.

### Taqman real-time quantitative PCR assay

The qPCR assay was performed in 20 μL reaction volume using Pro Taq HS Premix Probe real-time PCR Kit III (Accurate Biotechnology Co., Ltd, Hunan, China) according to the manufacturer’ s instructions. The primers (F3/R3) and probe (Probe-qPCR) for aPCR assay were listed in **Table 1**. In the 20 μL reaction mixture, there was 2.0 μL of extracted DNA template, 10 μL of 2 × Pro Taq HS Probe Premix III, 4.4 μL of DNase-free water, 1.0 μL of each primer F3/R3 (10 μM), 1.6 μL of Probe-qPCR (10 μM). Next, the reactions were examined on the Bio-rad CFX96 Touch Real-Time PCR Detection System (Bio-rad, Hercules, CA, USA). The qPCR procedure involved an initial denaturation step at a temperature of 95°C for a duration of 30 s, followed by 40 cycles of amplification (with a denaturation step at 95°C for 5 s and an annealing step at 60°C for 30 s). Every trial includes both a positive control (pEvpP plasmid) and a negative control.

### Specificity analysis

To assess the specificity of the RAA assay, eight additional bacterial pathogens were included: *P. fluorescens*, *L. adecarboxylata*, *V. rotiferianus*, *V. scophthalmi*, *V. alginolyticus*, *V. hyugaensis*, *H. piscium* and *V. azureus*. The genomic DNA of these bacteria were extracted as described above and then utilized as the template. The specificity test was performed under the aforementioned conditions and system, and repeated more than three times with each template and negative controls.

### Sensitivity analysis

To determine the sensitivity of the RAA assay, plasmid pEvpP was diluted in 10-fold serial dilutions ranging from 2.1×10^5^ to 2.1×10^0^ copies/μL, with six replicates for each dilution. For comparison, the same templates were tested in parallel using the qPCR assay. The sensitivity assays were carried out utilizing the aforementioned conditions and systems, and were repeated more than three times with each template and negative controls.

### Evaluation of the RAA assay using clinical samples

In order to assess applicability of the RT-RAA assay for *E. piscicida* detection in clinical and environmental samples, bacterial DNA were extracted from a total of 48 samples. Among these samples, 30 were clinical samples (24 positive and 6 negative), 9 were sea water samples (6 positive and 3 negative), and 9 were sediment samples (6 positive and 3 negative). Subsequently, the RAA assay was conducted on all 48 samples. For comparative purposes, the same samples were also subjected to the qPCR assay for *E. piscicida* in parallel.

### Statistical analysis

Probit analysis for the detection limit of the RT-RAA assay and qPCR assay was performed at a 95% probability level. The kappa and *p* values of the RT-RAA and qPCR assays were calculated. All statistical analysis was carried out with SPSS 21.0 (IBM, Armonk, NY).

## Results

### Primers and probe designed for the RT-RAA assay

Ten *EvpP* gene sequences of *E. piscicida* were obtained from the GenBank database. Based on the multiple sequence alignment analysis, conserved nucleotide residues were identified and visually emphasized with a black background. In order to design primers and probe, 10 *EvpP* sequences were aligned with F2, R2, and Probe-RAA primers. The sequence alignment revealed high conserved regions for primers and probe design (**Figure 1A**). After primer screening, the optimal primer pair F268–297/R401–430 was screened out according to the amplification curve and renamed F2/R2 (**Figure 1C**, **Table 1**).

### Specificity of RAA assay

**Figure 2:**
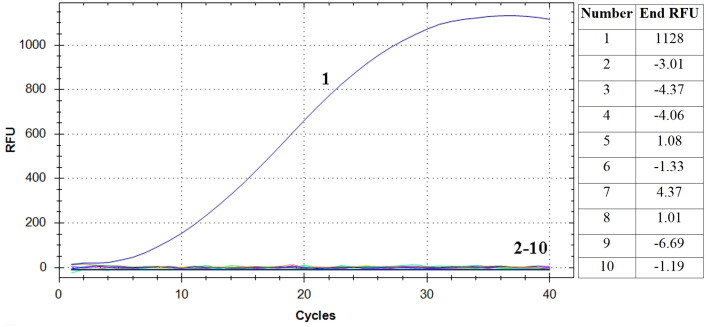
Specificity analysis of the RT-RAA assay. Only the reaction containing *E. piscicida* DC1 DNA as template produced a positive signal, while nucleic acid from all other eight bacteria and negative control did not. Curve 1: *E. piscicida*; Curve 2-10: *Pseudomonas fluorescens, Leclercia adecarboxylata, Vibrio rotiferianus, Vibrio scophthalmi, Vibrio alginolyticus, Vibrio hyugaensis, Haemophilus piscium and Vibrio azureus* and negative controls, respectively. The final relative fluorescence unit (RFU) values of each samples were shown in the right table.

The RAA assay showed a positive result for *E. piscicida* and negative results for *P. fluorescens*, *L. adecarboxylata*, *V. rotiferianus*, *V. scophthalmi*, *V. alginolyticus*, *V. hyugaensis*, *H. piscium* and *V. azureus* (**Figure 2**). The results showed that the established RT-RAA assay exhibited a considerable degree of specificity towards its intended targets, with no cases of cross-reactivity observed with other prevalent marine pathogenic bacteria.

### Analytical sensitivity of RT-RAA assay

**Figure 3:**
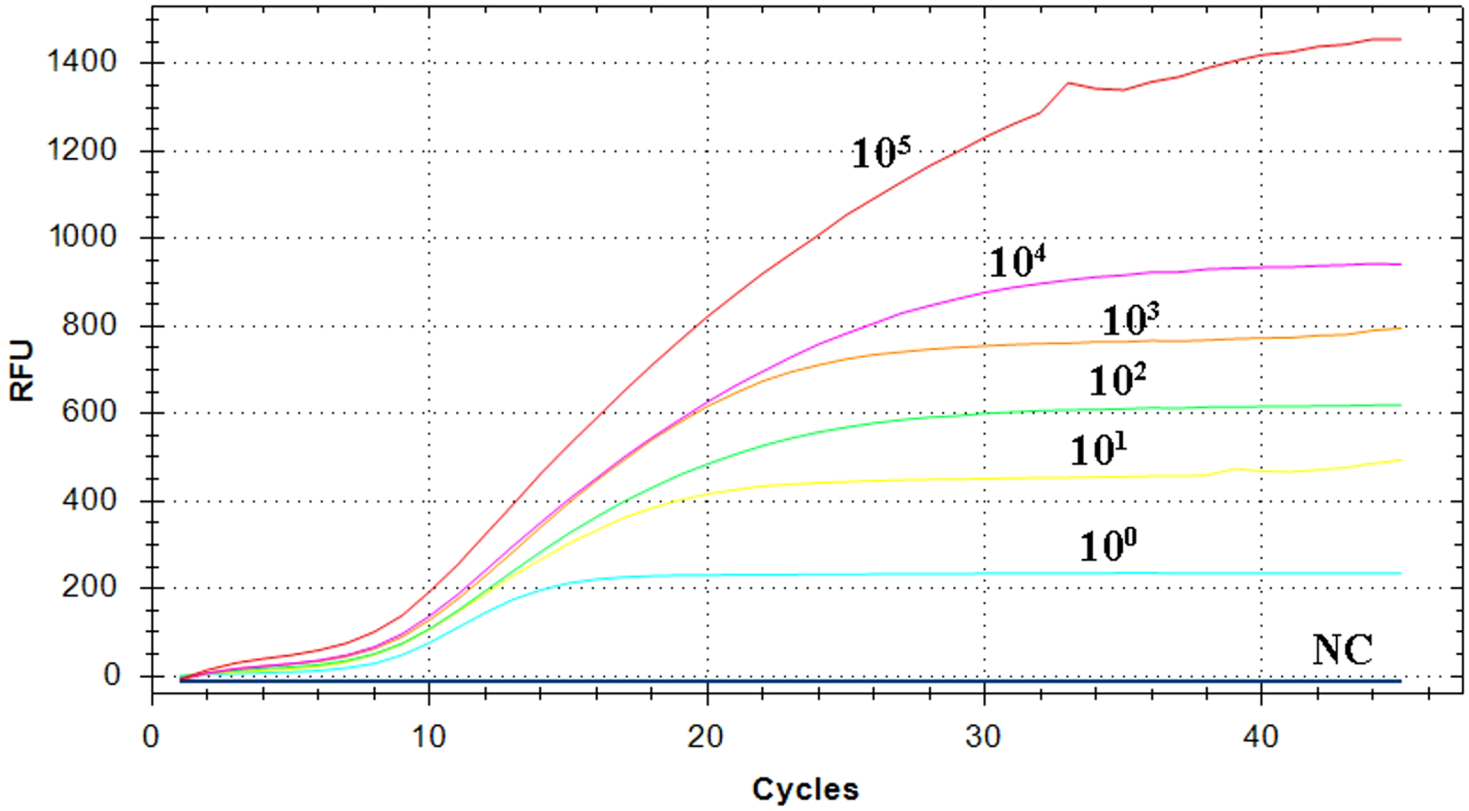
Sensitivity analysis of the RT-RAA assay. A panel of serially diluted recombinant plasmid pEvpP, from 10^5^ to 10^0^ copies per reaction and a negative control sample (NC), were used to determine the detection limit of the RT-RAA assay.

The pEvpP plasmid, which was serially diluted from 2.1×10^5^ to 2.1×10^0^ copies/μL, was subjected to testing using the RT-RAA assay. The results indicated that consistent signals were observed within the range of 2.1×10^0^ and 2.1×10^5^ copies/μL for each reaction. As a result, the detection limit of the RAA assay was determined to be 42 copies (0.14 fg) per reaction with a 95% probability (**Table 2**, **Figure 3**) (Probit analysis, *p*=0.015), which aligns with the detection limit of 42 copies per reaction for the qPCR method (**Table 2**, **Figure 3**) (Probit analysis, *p*=0.02).

**Table 2:** Assay data used for probit analysis to calculate the detection limits of RT-RAA and qPCR.

Copies/μL	Numbers of positive samples/six replicates of RT-RAA	Numbers of positive samples/six replicates of aPCR
2.1×10^5^	6/6	6/6
2.1×10^4^	6/6	6/6
2.1×10^3^	6/6	6/6
2.1×10^2^	6/6	6/6
2.1×10^1^	5/6	6/6
2.1×10^0^	1/6	0/6

### Evaluation of the RT-RAA assay using clinical and environmental samples

**Figure 4:**
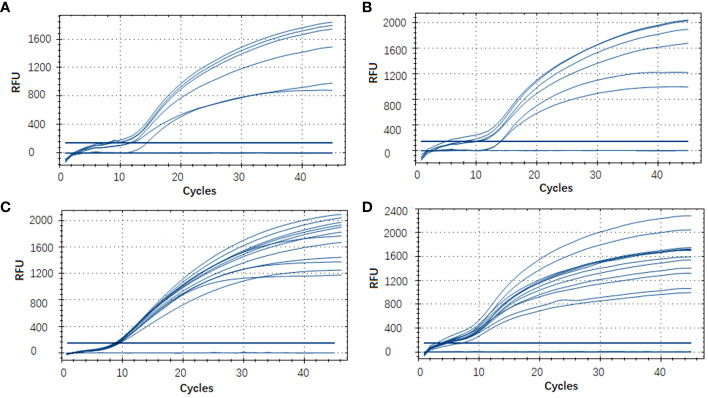
Evaluation of the RT-RAA assay using clinical and environmental samples. A total of 48 samples, consisting of 9 sea water samples **(A)**, 9 sediment samples **(B)**, 30 clinical fish samples **(C, D)** were analyzed with the RT-RAA assay.

In this RT-RAA assay, a total of 48 samples, consisting of 24 positive clinical samples, 6 positive sea water samples, 6 positive sediment samples, and 12 negative samples (6 clinical samples, 3 sea water samples, 3 sediment samples), were utilized, and the results were comparable with those obtained by qPCR. Using qPCR, 36 out of the 48 samples were identified as positive. The RT-RAA assay successfully detected and distinguished all 36 positive samples with complete accordance, sensitivity, and specificity (**Table 3**, **Figure 4**). Notably, no significant disparities were observed between the detection outcomes of the RT-RAA assay and qPCR. The RT-RAA assay yielded a kappa value of 1.0 (p < 0.001).

**Table 3:** Detection of *E. piscicida* DC1 in clinical samples.

Samples	qPCR	RT-RAA		Total	Performance of RT-RAA compared with qPCR		
		Positive	Negative		Accordance rate (%)	Kappa (κ)	p-value of kappa
Fish	Positive	24	0	24	100	1	< 0.001
	Negative	0	6	6	–	–	–
Sea water	Positive	6	0	6	100	1	< 0.001
	Negative	0	3	3	–	–	–
Sediment	Positive	6	0	6	100	1	< 0.001
	Negative	0	3	3	–	–	–
	Total	36	12	48	–	–	–

## Discussion

*E. piscicida*, a significant intracellular pathogen, is found extensively in aquatic surroundings and causes systemic infections in different species. Until now, several methods, including ELISA, ordinary PCR, qPCR, and QCM biosensor, have been employed for the detection of *E. piscicida* (Swain et al., 2001; Hong et al., 2009; Castro et al., 2010; Reichley et al., 2015). However, these methods pose challenges in their implementation in underfunded laboratories with limited resources and expertise. Currently, the detection of *E. piscicida* can be accomplished within 45 minutes using a relatively fast detection technique called loop-mediated isothermal amplification (LAMP) (Xie et al., 2013). Nevertheless, the current LAMP methods suffer from unwanted nonselective amplification, leading to significant background signals due to the increasing number of target sites. This nonspecific amplification significantly undermines the dependability of LAMP and imposes constraints on its utilization in clinical diagnostics (Hardinge and Murray, 2019). Therefore, it is crucial to establish a rapid and sensitive diagnostic method for clinical diagnosis of *E. piscicida*.

RAA is a novel isothermal amplification technique that has been developed in recent years. Compared with the general molecular detection methods, RAA possesses several advantages, such as low-cost, simplicity of operation (as it does not necessitate complex equipment or highly skilled personnel, can be conducted using portable device), low and constant reaction temperature (39°C), and rapid reaction speed (within 20 min) (Xie et al., 2012). As a result, RAA has been widely used in identifying viruses, bacteria, and parasites (Cui et al., 2022; Fu et al., 2022; Lin et al., 2022; Cui H et al., 2023; Cui X et al., 2023). Consequently, it represents a rapid and user-friendly method suitable for clinical use, particularly in primary laboratories.

Although the genomes of *E. piscicida* from various isolates exhibit a relatively conserved nature, genetic diversity can be observed among different strains. It is important to mention that sequence variations within the target region can significantly affect the detection results of molecular diagnostic techniques. Previous studies have identified 16S rRNA, *gyrB*, *hlyb*, and *EvpP* as potential targets for *E. piscicida* detection (Swain et al., 2001; Hong et al., 2009; Castro et al., 2010; Reichley et al., 2015). Among these, the *EvpP* gene is exclusive to *E. piscicida*, offering high specificity and diagnostic effectiveness. In the present study, the RT-RAA primers and probe were, therefore, designed based on the conserved region of *EvpP* gene.

Previous studies on viral and bacterial pathogens have indicated the detection limit of the RAA assay varied from 10 to 142 copies per reaction when tested against standard plasmids (Zhang et al., 2017; Cui et al., 2022; Fu et al., 2022; Lin et al., 2022; Cui H et al., 2023; Cui X et al., 2023). In this study, a novel and simple method for *E. piscicida* detection was proposed based on RAA technique. The well-established RT-RAA method exhibited excellent sensitivity, capable of detecting as few as 42 (0.14 fg) copies of standard plasmids per reaction, indicating a high level of sensitivity and consistent with the detection limit of the qPCR method. Additionally, specificity analysis confirmed that the established RT-RAA assay exhibited a notable level of specificity for its intended targets, as it did not exhibit any instances of cross-reactivity with other commonly found marine pathogenic bacteria. To evaluate the clinical feasibility of the RAA-based method, a total of 48 clinical and environmental samples were collected for *E. piscicida* detection. Both RT-RAA and qPCR techniques successfully identified 36 out of the 48 samples as positive, demonstrating a sensitivity and specificity of 100%. A strong agreement between the RT-RAA and qPCR assays was indicated by a κ value of 1.0 (p < 0.001). These findings indicate that the RT-RAA technique established in this research is highly appropriate for detecting *E. piscicida* infections in fish.

Taken together, in this study, we have successfully developed an RT-RAA assay that shows outstanding specificity and sensitivity. This assay offers a straightforward, expeditious, and dependable approach for detecting *E. piscicida*. The distinctive attributes of this RT-RAA assay render it particularly suitable for implementation in under-resourced diagnostic laboratories, thereby potentially facilitating prompt clinical detection and treatment in both clinical and onsite settings.

## Data availability statement

The datasets presented in this study can be found in online repositories. The names of the repository/repositories and accession number(s) can be found below: https://www.ncbi.nlm.nih.gov/genbank/, OR670479.1.

## Ethics statement

The animal study was approved by Ethics Committee of Yantai University. The study was conducted in accordance with the local legislation and institutional requirements.

## Author contributions

YD: Investigation, Methodology, Writing – original draft. DZ: Writing – original draft, Formal analysis, Software. BZ: Writing – original draft, Data curation, Resources. XX: Resources, Funding acquisition, Supervision, Validation, Writing – review & editing. JZ: Funding acquisition, Writing – review & editing, Conceptualization.
