# Supplementary material for: Development of a real-time recombinase-aided amplification assay for rapid and sensitive detection of Edwardsiella piscicida
Source: Front Cell Infect Microbiol. 2024 Mar 28;14:1355056. doi: 10.3389/fcimb.2024.1355056 (PMC11007066; doi:10.3389/fcimb.2024.1355056)
Supplement: Supplementary file 1 [file DataSheet_1.pdf]

Supplementary data

Table S1 Primers for screening.

Primer / probe	Sequence (5’- 3’)
F264-293	GTACGATGGCGGTCTGCGCCAGCGTATGCT
F265-294	TACGATGGCGGTCTGCGCCAGCGTATGCTG
F266-295	ACGATGGCGGTCTGCGCCAGCGTATGCTGA
F267-296	CGATGGCGGTCTGCGCCAGCGTATGCTGAT
F267-297	CGATGGCGGTCTGCGCCAGCGTATGCTGATC
F268-297	GATGGCGGTCTGCGCCAGCGTATGCTGATC
R401-430	TAAATCCACCGAACCAGGCATAGACGGGAA
R401-431	TTAAATCCACCGAACCAGGCATAGACGGGAA
R402-431	TTAAATCCACCGAACCAGGCATAGACGGGA
R403-432	CTTAAATCCACCGAACCAGGCATAGACGGG
R404-433	CCTTAAATCCACCGAACCAGGCATAGACGG
R405-434	CCCTTAAATCCACCGAACCAGGCATAGACG
